# Socio-economic and financial characteristics of companion animal clinics in Ankara Province, Türkiye: A cluster-based classification

**DOI:** 10.1371/journal.pone.0357364

**Published:** 2026-08-31

**Authors:** Arzu Peker, Lorenzo Serva, Yılmaz Aral, Şükrü Orkan, Oğuz Altın, Luisa Magrin

**Affiliations:** 1 Department of Animal Health Economics and Management, Faculty of Veterinary Medicine, Ankara University, Ankara, Türkiye; 2 Department of Animal Medicine, Production and Health, University of Padova, Viale dell’Università 16, Legnaro, Padova, Italy; 3 Department of Animal Health Economics and Management, Faculty of Veterinary Medicine, Dicle University, Diyarbakır, Türkiye; MOHME: Iran Ministry of Health and Medical Education, IRAN, ISLAMIC REPUBLIC OF

## Abstract

Companion animal clinics are an integral part of urban veterinary practice. Yet such clinics face several challenges, including rising operational costs, intense market competition, and shifting pet owners’ expectations. In contrast to previous studies focused on veterinary business models, little in-depth examination of determinants of clinic performance has been conducted to date. To address this gap, the current study evaluated the socio-economic and financial characteristics of companion animal clinics in Ankara Province, Türkiye, using Principal Component Analysis followed by k-means clustering. A cross-sectional survey was conducted among the owners or managers of 35 companion animal clinics using a questionnaire comprising 44 indicators. Cluster 1 (10 clinics, 28.57%), which included multi-service clinics, had the highest costs ($8616.77) and income ($10671.77) due to service diversity and high investments in staff and equipment. Cluster 2 (8 clinics, 22.86%) included clinics characterized by moderate costs and stable income, oriented toward efficiency and targeted services. Cluster 3 (8 clinics, 22.86%) included cost-efficient clinics that mainly provided cat care and achieved the highest profitability of $5093.65. Cluster 4 (9 clinics, 25.71%) included clinics specializing in the care of exotic animals, with the lowest monthly income of $5023.83 and difficulties in achieving regular profitability. The results of our study revealed that the differences between clinic profiles are generally related to operating cost structures and strategies, income sources, and the variety of services provided to clients. Clinics that manage operational costs in line with income streams and adapt to client needs tend to perform better financially. In conclusion, veterinary professionals can use these insights to improve their strategies, optimize resource use, and improve financial performance.

## 1. Introduction

In veterinary clinics, there is an interaction of many dynamics, including a client-focused culture. Today’s urban clinics face the need to meet changing client expectations while operating with limited resources, and therefore struggle to compete in various areas [1]. However, due to its significant contribution towards pet welfare and wellbeing, awareness of the business models is imperative for financial viability and service development. Successful practice in veterinary care is a combination of medical skill and additional professionally-related competencies, including communications, interpersonal skills, and business management skills [2].

In recent years in Türkiye, the number of private veterinary clinics operating for pets has been rapidly increasing and becoming widespread, especially in metropolitan cities such as Ankara, Istanbul and Izmir [3]. As one of the largest urban centers in Türkiye, Ankara has been witnessing a consistent increase in pet ownership, accompanied by increased awareness of animal welfare, changing social dynamics and increased demand for pets as a result of COVID-19 [4,5]. The nature of city apartment living and modern lifestyles created an environment in which people preferred cats and even small dog breeds as companion animals [6,7].

According to Chamber of Veterinary Surgeons Ankara Region, there are around 528 veterinary clinics (including companion animal, farm animal and mixed clinics) actively working in the region [8]. Veterinary clinics offer not only routine veterinary services but also offer diagnostics, pet hoteling, grooming and some speciality services [9]. However, some veterinarians have outdated views of their business model and believe that offering more complex services will provide them with financial and revenue advantages [10].

Although the industry is growing overall, veterinary clinics face numerous challenges. With high operating costs, intense competition, and constantly changing client demands [11], clinics are under considerable pressure to optimize their services in response to the market. For meaningful development of strategies by clinicians and policymakers for sustainable growth and better service delivery in the sector, these challenges must be understood through evidence-based knowledge of the socio-economic and financial dynamics peculiar to veterinary practices.

Few prior studies have examined veterinary clinics in terms of economic and management aspects [1,3,6,11,12], but none have comprehensively combined socio-economic and financial data to identify socio-economic patterns among veterinary clinics. This study represents the first application of the k-means clustering approach to veterinary clinics by exploring their socio-economic and financial characteristics. Our study aims to identify k-means clusters based on socio-economic focus and financial performance by analyzing data from companion animal clinics in Ankara Province, Türkiye.

This study, therefore, contributes to the literature on veterinary economics by providing a comprehensive framework that helps in understanding the financial and operational dynamics of the clinics that deal with companion animals.

## 2. Materials and methods

### 2.1. Selected veterinary clinics, study areas and structure of the questionnaire

The study included data obtained from the cross-sectional survey conducted with 35 veterinary clinics (specialized in companion animals) located in Ankara province of Türkiye.

A set of 44 indicators, important for representing clinics in socio-economic and financial analyses based on previous literature findings [6,11], formed the basis of the questionnaire, which was then used to interview 35 veterinary clinics. The participating companion animal clinics were recruited through voluntary participation. Because no comprehensive official registry of all eligible companion animal clinics operating in Ankara Province was available during the study period, the total target population could not be determined. Consequently, neither an exact response rate nor the sampling proportion could be calculated. Before conducting the surveys, a written Informed Consent Form was provided to each clinic owner, outlining the purpose, procedures, and confidentiality measures of the study. The surveys were conducted between 10.06.2019 and 19.02.2020, with each interview lasting approximately 60 minutes. Through in-person interviewing, both multi-choice and open-ended questionnaires have been utilized. All collected data were fully anonymized, and confidentiality was strictly maintained. This study did not involve the collection or use of medical records or archived samples, and at no point did the researchers have access to any personally identifiable information of the participants.

Tables 1 and 2 below present first variables—qualitative and quantitative, respectively—that act in statistics analysis as indicators. Five important financial metrics—profit margin, cost-to-income ratio, labour productivity, profit-per-patient, and asset turnover ratio—have been used to evaluate financial wellbeing and operational effectiveness for the clinics.

**Table 1:** General characteristics of the veterinary clinics based on the qualitative variables.

		*Total*	
*Variables*	*Classes*	*N*	*%*
***Socio-Economic Variables***			
Gender	Male	24	68.57
	Female	11	31.43
Specialities	Ph.D	6	17.14
	Master	17	48.57
	*Others (training, seminar, congress etc.)*	12	34.29
District	Altındağ	2	5.70
	Çankaya	20	57.10
	Etimesgut	1	2.90
	Gölbaşı	1	2.90
	Keçiören	5	14.30
	Yenimahalle	6	17.10
**Technical Variables**			
Client record software	Yes	30	85.71
	No	5	14.29
Staff performance following system	Yes	24	68.57
	No	11	31.43
Pricing based on association tariff	Yes	23	65.71
	No	12	34.29
Service to home	Yes	19	54.29
	No	16	45.71
7/24 open	Yes	14	40.00
	No	21	60.00
Emergency/ambulance service	Yes	8	22.86
	No	27	77.14
Pet hotel existence	Yes	21	60.00
	No	14	40.00
Pet products selling	Yes	31	88.57
	No	4	11.43
Variety of services	Yes	26	74.29
	No	9	25.71
Using social media	Yes	23	65.71
	No	12	34.29
Following financial status	Yes	18	51.43
	No	17	48.57
Stock control	Yes	16	45.71
	No	19	54.29
Working out of hours	Yes	28	80.00
	No	7	20.00

**Table 2:** General characteristics of the veterinary clinics based on the quantitative variables.

*Variables*	*Minimum*	*Maximum*	*Mean, SD*
**Socio-Economic Variables**			
Actively working (years)*	4.00	31.00	10.43 ± 7.10
Cat patient (%) *	40.00	90.00	62.21 ± 12.04
Dog patient (%) *	10.00	50.00	31.90 ± 10.19
Exotic patient (%) *	0.00	30.00	6.00 ± 6.88
Number of staff *	1.00	8.00	3.37 ± 1.68
Weekly working hours *	42.00	114.00	69.91 ± 16.83
Active animal number *	40.00	4500.00	627.23 ± 855.16
Current Investment Value ($) *	9463.72	141955.84	60973.41 ± 28932.24
Equipment Value ($) *	1261.83	86750.79	33271.74 ± 20652.26
**Monthly Income Factors ($)**			
General Examination Income *	126.18	4810.73	1980.24 ± 1115.34
Lab Income *	0.00	1892.74	691.98 ± 499.65
Diagnostic Image Income *	0.00	2602.52	649.26 ± 641.30
Operation Income *	0.00	4022.08	1408.97 ± 912.69
Vaccination- Antiparasitic Income *	189.27	5205.05	1871.34 ± 1104.58
Petshop (including pet food sales) Income *	0.00	3785.49	851.67 ± 867.89
Pet Grooming Income *	0.00	2365.93	527.96 ± 518.31
Pet Hotel Income *	0.00	2819.12	455.61 ± 639.17
Monthly Average Income *	630.91	18927.44	8437.02 ± 3885.57
Monthly Average Profit *	−4203.47	6987.38	2443.90 ± 2462.54
**Monthly Cost Factors**			
Vaccination-Medicine Cost (% of total) *	10.00	65.09	30.28 ± 13.66
Rent Cost (% of total) *	0.00	38.46	12.32 ± 8.34
Labour Cost (% of total) *	0.00	50.96	23.88 ± 14.08
Equipment-Material Cost (% of total) *	1.00	43.42	11.09 ± 10.59
Other Costs (% of total) *	4.00	40.59	22.43 ± 8.78
Monthly Average Cost ($) *	820.19	18309.62	5993.12 ± 3587.98

* Variables used for the principal component analysis.

** (Average exchange rate for 2019–2020: US$1 = 6,34 TL).

For each of these five metrics, tertile segments have been developed for the clinics.

### 2.2. Calculation of financial performance parameters

1. **Net Profit Margin:** It can also be defined as the relation of net profit to total revenues, usually expressed in terms of percentage. The profit margin indicates the dollar amount, from each dollar of revenue, that the clinic retains as profit [13].
2. **Cost-to-Income Ratio:** This is the ratio of a clinic’s cost to its income that assesses its operating efficiency. The lower the ratio, the better is the efficiency. A lower ratio means the clinic can have more of the revenue turned into profit. It was calculated as: [14].
3. **Labor productivity:** Labor productivity refers to productivity of the clinic’s staff in relationship to income generated. Labor productivity is defined as total revenues divided by full-time equivalent (FTE) employees. This would indicate how well workforce is utilized to generate revenue for the clinic [15].
4. **Profit/Patient Ratio:** It could be calculated as profit per patient: the overall profit divided by the overall number of patients treated within the period yields the amount of money each patient interaction brings into the clinic [16].
5. **Asset Turnover Ratio (ATR):** This ratio reflects how well the clinic is using its assets in generating revenues. The high asset turnover ratio depicts that clinics derive revenues from the asset base. It was calculated as: [17].

### 2.3. Statistical analysis

Following the import of the questionnaire data into Excel® LTSC MSO 2021, the missing or anomalous values for each original variable were analyzed. The statistical analysis was conducted using SAS/ STAT (Inst.Inc., Cary, NC), XLSTAT (Addinsoft, New York, NY), and IBM SPSS 22.0 software.

In a complex dataset analysis, efficient feature selection enhances a model’s predictive power while reducing complexity [18,19]. Principal Component Analysis (PCA) offers an unsupervised approach to reduce a dataset’s dimensionality by extracting fewer orthogonal latent variables, thereby simplifying the structure of the original data. Additionally, PCA provides an effective means to transform data for easier clustering through other techniques like k-means. However, it is important to note that PCA accounts only for the relationships between predictors without incorporating classification factors. By applying an orthogonal transformation, PCA reduces the pool of correlated variables into a smaller set of uncorrelated components, known as Principal Components (PCs) or orthogonal latent variables. This method increases data interpretability with minimal information loss [20] which may mitigate some noise effects or redundant information.

In this study, a multivariate aggregation approach was implemented in three consecutive steps: (1) PCA for dimensionality reduction, (2) k-means clustering of the clinics based on the extracted PCs, and (3) statistical evaluation of k-means clusters using Analysis of Variance (ANOVA) or contingency table tests (e.g., χ² test). Flowchart of the statistical framework can be seen in S1 Fig.

#### 2.3.1. Principal component analysis (PCA).

To mitigate the impact of multicollinearity, all linear dependent variables were assessed prior to analysis, and variables showing excessive correlation or redundancy were excluded from the dataset and are not presented in this study. A correlation matrix was subsequently calculated, and indicators exhibiting high correlations (r > 0.6) were removed and not presented in this study.

PCA was applied to the remaining quantitative original variables, ensuring the overall Kaiser–Meyer–Olkin (KMO) measure of sampling adequacy exceeded 0.5. This step ensured the suitability of the dataset for PCA. The method separated the communality (proportion of variance explained by common factors) from uniqueness (proportion of variance not shared with other variables, calculated as 1 − communality). Greater uniqueness indicates that the variable contributes less to the shared factor model [21]. Uniqueness includes random error combined with the specific variance of the variable, which cannot be disentangled [22,23].

A “varimax” rotation was applied to the standardized variables (centered and scaled) to facilitate the interpretation of the extracted components [24]. Components were selected based on the Kaiser criterion (eigenvalues > 1) [25], while others were discarded from further calculations. Variables were considered strongly associated with a component if their absolute loading values exceeded 0.50 [22,23]. The Principal Components (PCs) were named according to the most influential original variables to enhance data interpretation. Specifically, PCs were assigned descriptive labels based on the most representative original variables, considering only those with factor loadings greater than 0.5. To ensure a robust interpretation of the orthogonal latent variables, only PCs with at least two original variables exhibiting factor loadings above this threshold were retained. This reduction in the number of latent variables facilitates a more concise and meaningful discussion of the results.

#### 2.3.2. Clinics K-means clustering.

A k-means clustering algorithm was applied to the extracted PCs to identify homogeneous groups among clinics and highlight their similarities. The optimal number of k-means clusters (K) was determined using the silhouette score, which measures the cohesion within k-means clusters and the separation between them [26]. However, to ensure meaningful k-means clustering, each k-means cluster contained a minimum of eight clinics.

#### 2.3.3. K-means cluster explanation using analysis of variance (ANOVA) and contingency table tests.

For continuous variables, normality was assessed using the Shapiro–Wilk test, visual inspection of frequency distributions, and Q-Q plots (quantile-quantile plots). A total of 43 omnibus tests were conducted: 5 one-way ANOVAs for the retained principal component scores, 24 one-way ANOVA for continuous variables, and 14 global contingency-table tests for categorical or supplementary variables. Post hoc pairwise comparisons following significant ANOVA results were adjusted using the Bonferroni method. Associations between cluster membership and categorical variables were assessed using Pearson’s χ^2^ test or Fisher’s exact test for r × c contingency tables, as appropriate. When a significant global association was detected, cellwise Fisher’s exact tests were used to identify cells with observed frequencies significantly higher or lower than those expected under independence. Because the cluster comparisons were exploratory, the omnibus P values were reported without adjustment across the complete set of outcomes. Therefore, the increased risk of Type I error associated with multiple testing was explicitly acknowledged, and the cluster comparisons are considered exploratory and hypothesis-generating rather than confirmatory. Statistical significance was defined as P ≤ 0.05.

A post hoc power analysis was conducted for the statistically significant omnibus tests using G*Power, version 3.1 [27].

#### 2.3.4. Ethics statement.

The study involved anonymous face-to-face interviews with owners or managers of companion animal clinics. Participation was entirely voluntary, and written informed consent was obtained from all participants prior to data collection. No personal or sensitive data were collected, and all responses were anonymized before analysis. According to the applicable institutional and national regulations in force at the time of the study, formal ethics committee approval was not required for this type of anonymous voluntary survey.

## 3. Results

### 3.1. General veterinary clinic characteristics and current problems

The general characteristics of the veterinary clinics based on qualitative and quantitative variables are presented in Table 1 and Table 2, respectively. The average working year of pet clinics was 10.43. The clinic owners’ specializations are mostly at the Master’s degree level (48.57%). The percentage of cat patients (61.21%) are relatively higher than the percentage of dog patients (31.90%). The average number of staff working in the veterinary clinics are 3.37. When the monthly income factors are examined; it was seen that general examination income ($1980.24) has the highest share and followed by vaccination income ($1871.34), operation income ($1408.97) and pet shop income ($851.67) respectively. Vaccination-medicine cost (30.28%) has the highest ratio among monthly cost factors in veterinary clinics. Then it was followed by labour cost (23.88%), other costs (22.43%) (such as insurance, accounting, credit interest, water/electricity, administrative and communication costs etc.) and equipment-material cost (11.09%) respectively. While most of the clinics are located in the Çankaya district (57.10%) it is followed by the Yenimahalle district (17.10%) and both districts have a high population and a high demand for pet services.

The Table 3 presents the main operational difficulties experienced by clinics in the order of significance measured from the survey responses. According to the survey results, unfair competition among clinics remain the greatest concern, with 31.40% of the participants rating this concern as their most important and 20% marking it as their second and third most important concerns. Problems with the clients also took a large portion of the clinics, being named the main concern by 14.30% of respondents, second by 8.60%, and third by 22.90%. Furthermore, price increases in vaccines, medicines, and material supplies were the most pressing issues for 11.40% and the second issue for 22.90%. Other challenges pertained to inability to provide qualified staffs, which was rated the second most serious issue by 14.30% and third by 17.10%. Focusing on the issue of professional worthlessness, 14.30% cited this as a priority as well as concerns regarding uncollectible receivables.

**Table 3:** Current problems faced by veterinary clinics.

Current Problems	Order of Importance		
	1 (%)	2 (%)	3 (%)
Problems and price increases in vaccine-medicine-material supply	11.40	22.90	8.60
Unfair competition between clinics	31.40	20.00	20.00
Low number of patients and low income	5.70	0.00	2.90
Problems with clients	14.30	8.60	22.90
Low recognition	0.00	5.70	2.90
Uncollectible receivables	8.60	14.30	5.70
Financial management issues	2.90	2.90	8.60
Inability to provide qualified staff	8.60	14.30	17.10
Low profit margin	0.00	2.90	2.90
Professional worthlessness	14.30	5.70	5.70
Ethical values	0.00	2.90	0.00
Medicine Tracking System (MTS)	2.90	0.00	2.90
**Current Problems**	**Order of Importance**		
	**1 (%)**	**2 (%)**	**3 (%)**
Problems and price increases in vaccine-medicine-material supply	11.40	22.90	8.60
Unfair competition between clinics	31.40	20.00	20.00
Low number of patients and low income	5.70	0.00	2.90
Problems with clients	14.30	8.60	22.90
Low recognition	0.00	5.70	2.90
Uncollectible receivables	8.60	14.30	5.70
Financial management issues	2.90	2.90	8.60
Inability to provide qualified staff	8.60	14.30	17.10
Low profit margin	0.00	2.90	2.90
Professional worthlessness	14.30	5.70	5.70
Ethical values	0.00	2.90	0.00
Medicine Tracking System (MTS)	2.90	0.00	2.90

### 3.2 Results of principal component analysis

After performing the PCA on the original 25 variables, only 5 PCs with at least two original variables exhibiting factor loadings above 0.5 threshold were retained. Component 1 explained 28.66% of variance and all 5 principal components explained ca. 64.22% of the variance (Table 4).

**Table 4:** Eigenvalues of the five principal components and percentages of explained variance.

Principal Components	Initial eigenvalues		
	Eigenvalue	% of variance	Cumulative % of variance
PC1	7,45	28.66	28.66
PC2	2,82	10.84	39.50
PC3	2,58	9.92	49.42
PC4	2,18	8.39	57.81
PC5	1,67	6.41	64.22

Factor loadings for each variable and each indicator on axes are given in Table 5. Each principal component (PC) was named based on the dominant characteristics it represented. The highest eigenvector weights for PC1 (Multi-Service clinics) were number of staff, current investment and equipment value, monthly average cost, various incomes (lab, operation, diagnostic image, vaccination/antiparasitic, petshop, pet hotel, pet groomer) and monthly average income. The highest weights for PC2 (Experienced cat centre clinics) were the percentage of cat patients (%) and actively working years. For PC3 (Cost-efficient dog centre clinics) the highest weights were percentage of dog patients (%), monthly average profit, vaccination-medicine cost (% of total) and weekly working hours. For PC4 (Cost-conscious general care clinics) the highest weight was general examination income and other costs (% of total). The highest weight for PC5 (Niche exotic animal centre clinics) was the percentage of exotic patients (%) and actively working years.

**Table 5:** Factor loadings for the first 5 latent variables for each of the variables according to principal component analysis.

Variables	Principal Component				
	PC1	PC2	PC3	PC4	PC5
Actively working (years)	0.120	**0.651**	0.010	0.131	**0.505**
Cat patient (%)	−0.002	**0.728**	−0.496	−0.157	−0.356
Dog patient (%)	0.208	**−0.541**	**0.687**	0.257	−0.036
Exotic patient (%)	−0.297	−0.472	−0.137	−0.106	**0.668**
Number of staff	**0.755**	−0.039	0.176	0.318	0.087
Weekly working hours	−0.190	−0.298	**−0.555**	−0.157	0.243
Current Investment Value	**0.659**	0.366	0.030	−0.174	0.180
Equipment Value	**0.715**	0.255	−0.041	0.022	0.306
Equipment-Material Cost (% of total)	0.139	−0.011	0.364	−0.479	−0.077
Vaccination-Medicine Cost (% of total)	−0.396	−0.407	**−0.531**	0.001	−0.004
Rent Cost (% of total)	−0.364	−0.358	−0.301	0.379	−0.118
Other Costs (% of total)	0.075	0.284	0.171	**−0.545**	0.283
Monthly Average Cost	**0.792**	−0.255	0.190	−0.296	−0.203
General Examination Income	0.338	−0.240	0.023	**0.501**	0.127
Lab Income	**0.695**	−0.358	−0.005	−0.061	0.013
Diagnostic Image Income	**0.652**	−0.062	−0.174	−0.274	0.263
Operation Income	**0.764**	−0.021	−0.240	0.027	−0.144
Vaccination- Antiparasitic Income	**0.509**	0.158	−0.293	0.206	−0.283
Petshop (including pet food) Income	**0.616**	−0.190	−0.153	−0.233	−0.353
Pet Groomer Income	**0.583**	−0.248	−0.256	−0.369	−0.183
Pet Hotel Income	**0.565**	−0.245	−0.030	−0.205	0.266
Monthly Average Income	**0.927**	−0.201	−0.236	0.021	−0.093
Monthly Average Profit	0.308	0.055	**−0.649**	0.464	0.150

Factor loadings > 0.5 have been typed bold.

*PC1: Multi-service clinics, PC2: Experienced cat centre clinics, PC3: Cost-efficient dog centre clinics, PC4: Cost-conscious general care clinics, PC5: Niche exotic animal centre clinics.

### 3.3. Results of k-means clustering

K-means cluster analysis has been performed to establish the typology of the pet clinics. K-means clustering identified four groups of clinics. Cluster 1 consisted of 10 clinics, Cluster 2 of 8 clinics, Cluster 3 of 8 clinics and Cluster 4 of 9 clinics. In Table 6, the mean principal component scores for the five principal components across k-means clusters and comparisons between clusters were presented. K-means cluster interpretations were based on the dominant principal component scores together with the descriptive operational and financial characteristics of the clinics within each cluster. Therefore, cluster descriptions reflect multidimensional profiles rather than a single principal component.

**Table 6:** Mean scores of the five retained principal components across the four k-means clusters.

K-means clusters	Multi-service Clinics	Experienced cat centre clinics	Cost-efficient dog centre clinics	Cost-conscious general care clinics	Niche exotic animal centre clinics
1	**3,121** ^a^	0,081	1,197^a^	−1,300	0,483
2	−0,579^ab^	−1,091	**1,536** ^a^	0,126	−0,761
3	−1,537^ab^	**1,498**	−2,034^c^	−0,079	−0,869
4	−2,313^b^	−0,990	−0,340^b^	0,550	**1,058**
***P***	**0,005**	**0,040**	**0,0002**	0,065	**0,048**

Within each principal component, cluster means with different lowercase superscript letters differ significantly according to Bonferroni-adjusted pairwise comparisons (P ≤ 0.05). Values in bold indicate the dominant principal component scores used to support the interpretation of each cluster.

Table 7 shows descriptive statistics of quantitative variables for each of 4 k-means clusters. Table 8 includes frequencies of qualitative variables also by each k-means cluster.

**Table 7:** Means ± standard errors for continuous variables in the 4 k-means clusters and comparisons between them.

Variables	K-means cluster 1 (n = 10)		K-means cluster 2 (n = 8)		K-means cluster 3 (n = 8)		K-means cluster 4 (n = 9)		P
	Mean	SE	Mean	SE	Mean	SE	Mean	SE	
Actively working (years)*	9.10	2.09	7.88	2.34	6.63	2.34	15.78	2.21	*0,064*
Cat patient (%)	53.45^b^	3.43	61.63^a,b^	3.84	66.88^a,b^	3.84	68.33^a^	3.62	***0,024***
Dog patient (%)	41.65^a^	2.22	35.13^a,b^	2.48	26.88^b,c^	2.48	22.67^c^	2.34	***<.0001***
Exotic patient (%)	4.90	2.19	3.88	2.45	6.25	2.45	8.89	2.31	*0,472*
Number of staff	5.30^a^	0.369	2.50^b^	0.412	3.00^b^	0.412	2.33^b^	0.388	***<.0001***
Weekly working hours	61.00	4.987	68.88	5.576	81.75	5.576	70.22	5.257	*0,071*
Current Investment Value ($)	66798.11	9465.00	61119.87	10582.20	54416.40	10582.20	60199.79	9976.99	*0,856*
Equipment Value ($)	42981.07	6516.53	28489.75	7285.70	29081.23	7285.70	30459.17	6869.03	*0,384*
Equipment-Material Cost (% of total)	10.97^a,b^	2.829	21.60^a^	3.163	4.44^b^	3.163	7,81^b^	2.982	***0,004***
Vaccination-Medicine Cost (% of total)	23.19^b^	3.726	24.60^b^	4.165	42.78^a^	4.165	32.10^a,b^	3.927	***0,007***
Rent Cost (% of total)	12.22^a,b^	2.317	6.64^b^	2.590	19.61^a^	2.590	11.00^a,b^	2.442	***0,011***
Labour Cost (% of)	31.60	4.304	23.93	4.812	17.49	4.812	20.95	4.537	***0,168***
Other Costs (% of total)	22.02^a,b^	2.513	23.24^a,b^	2.810	15.69^b^	2.810	28.14^a^	2.649	***0,027***
Monthly Average Cost ($)	8616.77^a^	943.79	6900.04^a,b^	1055.19	5178.43^a,b^	1055.19	2995.97^b^	994.84	***0,002***
General Examination Income ($)	2496.06	329.90	1910	368.84	2262.42	368.84	1218.72	347.75	*0,068*
Lab Income ($)	1029.18^a^	134.29	706.82^a,b^	150.15	749.21^a,b^	150.15	253.24^b^	141.56	***0,004***
Diagnostic Image Income ($)	867.51	205.35	420.44	229.58	604.30	229.58	650.11	216.45	*0,547*
Operation Income ($)	1800.08	271.87	1269.22	303.96	1709.38	303.96	831.58	286.58	*0,083*
Vaccination- Antiparasitic Income ($)	2070.19^a,b^	318.58	1673.40^a,b^	356.18	2632.10^a^	356.18	1150.11^b^	335.81	***0,033***
Petshop (including pet food) Income ($)	958.20	261.03	990.73	291.84	1234.23	291.84	269.63	275.15	*0,108*
Pet Grooming Income ($)	645.35	164.66	510.65	184.09	653.59	184.09	301.26	173.57	*0,454*
Pet Hotel Income ($)	805.21	195.88	167.09	219.00	426.85	219.00	349.19	206.47	*0,180*
Monthly Average Income ($)	10671.77^a^	1026.45	7648.36^a,b^	1147.61	10272.08^a^	1147.61	5023.83^b^	1081.97	***0,003***
Monthly Average Profit ($)	2055.00^b^	632.30	748.32^b^	706.94	5093.65^a^	706.94	2027.87^b^	666.50	***0,001***

* Variables used for the principal component analysis.

** (Average exchange rate for 2019–2020: US$1 = 6,34 TL).

Within each each variable, cluster means with different lowercase superscript letters differ significantly according to Bonferroni-adjusted pairwise comparisons (P ≤ 0.05).

**Table 8:** Categorical variables in the 4 k-means clusters and comparisons between them.

Variables	Classes	K-means cluster 1 (n = 10)		K-means cluster 2 (n = 8)		K-means cluster 3 (n = 8)		K-means cluster 4 (n = 9)		Total (n = 35)		P
		*N*	*%*	*N*	*%*	*N*	*%*	*N*	*%*	*N*	*%*	
Client record software	Yes	10	100	7	87.50	7	87.50	6	66.60	30	85.70	*0,224*
	No	0	0	1	12.50	1	12.50	3	33.30	5	14.20	
Pet hotel existence	Yes	9	90.00	5	62.50	3	37.50	4	44.40	21	60.00	*0,095*
	No	1	10.00	3	37.50	5	62.50	5	55.60	14	40.00	
Pet products selling	Yes	9	90.00	7	87.50	7	87.50	8	88.90	31	88.60	*0,998*
	No	1	10.00	1	12.50	1	12.50	1	11.10	4	11.40	
Variety of services	Yes	8	80.00	5	62.50	5	62.50	8	88.90	26	74.30	*0,505*
	No	2	20.00	3	37.50	3	37.50	1	11.10	9	25.70	
Using social media	Yes	6	60.00	6	75.00	6	75.00	5	55.60	23	65.70	*0,760*
	No	4	40.00	2	25.00	2	25.00	4	44.40	12	34.30	
Following financial status	Yes	7	70.00	6	75.00	3	37.50	2	22.20	18	51.40	*0,077*
	No	3	30.00	2	25.00	5	62.50	7	77.80	17	48.60	
Stock control	Yes	9	90.00	5	62.50	4	50.00	5	55.60	16	45.70	*0,268*
	No	1	10.00	3	37.50	4	50.00	4	44.40	19	54.30	
Working out of hours	Yes	9	90.00	8	100	6	75.00	5	55.60	28	80.00	*0,106*
	No	1	10.00	0	0.00	2	25.00	4	44.40	7	20.00	
District	Altındağ	0	0.00	1	12.50	0	0.00	1	11.11	2	5.70	
	Çankaya	6	60.00	5	62.50	4	50.00	5	55.56	20	57.10	
	Etimesgut	0	0.00	0	0.00	1	12.50	0	0.00	1	2.90	*0,068*
	Gölbaşı	0	0.00	0	0.00	1	12.50	0	0.00	1	2.90	
	Keçiören	0	0.00	2	25.00	0	0.00	3	33.33	5	14.30	
	Yenimahalle	4	40.00	0	0.00	2	25.00	0	0.00	6	17.10	
**Tertiles**												
Profit margin	1	5	50.00	4	50.00	0	0.00^↓^	3	33.30	12	34.30	***0,001***
	2	5	50.00	4	50.00	1	12.50	1	11.10	11	31.40	
	3	0	0.00^↓^	0	0.00^↓^	7	87.50^↑^	5	55.60	12	34.30	
Cost/Income ratio	1	0	0.00^↓^	0	0.00^↓^	7	87.50^↑^	5	55.60	12	34.30	***0,001***
	2	5	50.00	4	50.00	1	12.50	1	11.10	11	31.40	
	3	5	50.00	4	50.00	0	0.00^↓^	3	33.30	12	34.30	
Labor productivity	1	4	40.00	1	12.50	0	0.00	3	33.30	121	22.90	*0,065*
	2	5	50.00	2	25.00	3	37.50	5	55.60	1	42.90	
	3	1	10.00	5	62.50	5	62.50	1	11.10	12	34.20	
Profit/patient ratio	1	4	40.00	4	50.00	1	12.50	3	33.30	12	34.30	*0,138*
	2	5	50.00	2	25.00	1	12.50	3	33.30	11	31.40	
	3	1	10.00	2	25.00	6	75.00	3	33.30	12	34.30	
Asset Turnover ratio (ATR)	1	4	40.00	1	12.50	1	12.50	6	66.70	12	34.30	*0,106*
	2	2	20.00	5	62.50	3	37.50	1	11.10	11	31.40	
	3	4	40.00	2	25.00	4	50.00	2	22.20	12	34.30	

Upward (↑) and downward (↓) symbols indicate cell frequencies significantly higher or lower, respectively, than those expected under independence, according to cellwise Fisher’s exact tests at P ≤ 0.05.

***K-means Cluster 1 (n = 10, 28.57% of the clinics).*** Cluster 1 which is defined with a focus on delivering a variety of services, demonstrates the highest percentage of dog patients with the ratio of 41.65% and the greatest monthly average cost with $8616.77. The clinics have been reported to employ the largest teams among k-means clusters (average 5.30 staff) and make good investment in equipment. Additional details show that these clinics also record the highest monthly average income which is $10671.77, with a significant contribution of laboratory income with $1029.18. All clinics belonging to Cluster 1 utilizes a client records software, showing a significant emphasis on administration and management of clients. Social media utilization is in a moderate rate of engagement with 60% of the clinics using it.

Clinics from this k-means cluster are equally distributed in the first two groups with a rate of 50% in terms of their profit margin. When it comes to cost-to-income ratios, they are evenly split, with 50% in the middle and highest level. For the Cluster 1, 50% of clinics working within the middle labor productivity framework.

***K-means Cluster 2 (n = 8, 22.86% of the clinics).*** Cluster 2 seems to have important volume of cat patients with the ratio of 61.63%. With smaller teams (2.50 staff on average) these clinics are cost effective despite having a higher monthly average income of $7648.36. The adoption of client record software (87.50%) and pet product sales (87.50%) are high in the Cluster 2 clinics. These clinics show significant investment in equipment-material costs with the ratio of 21.60%. The level of social media usage in this group is 75%. However, only 75% track their financial status, and 62.50% follow stock control practices.

In Cluster 2, both the profit margin and the cost to income ratio demonstrate an evenly spread distribution across tertiles with no less than 50% for all the indicators’ mid-tertiles. The labor productivity is high with 62.50% of the sample in the third tertile. In terms of ATR, 87.50% of the clinics are located in tertiles II and III.

***K-means Cluster 3 (n = 8, 22.86% of the clinics).*** Cluster 3 has a significant emphasis on cat patients with the ratio of 66.88% and follows a low monthly average cost structure among k-means clusters with $5178.43. They are more profitable in terms of income, having averaged a net profit of $5093.65 per month. Even though their team is considerably smaller (3 employees on average), Cluster 3 focuses on income generation through sales of ancillary services like pet products or vaccinations. In Cluster 3, utilization levels of client record software and pet product sales usage reveal similar patterns, about 87.50%.

This k-means cluster stands out in terms of financial performance, given that 87.50% of the clinics fall within the highest third of the profit margin and 50% for ATR hence supporting their low operating costs and relatively high profit as compared to income. Labor productivity is 62.50% also being in the top tertile.

***K-means Cluster 4 (n = 9, 25.71% of the clinics).*** These clinics have the lowest monthly average cost per month with $2995.97 and monthly average income with $5023.83 and have greater exotic animals share (%) than other gropus. Cluster 4’s operational strategy is quite low comparing with other k-means clusters and employs a smaller team of workers (2.33 on average) and offers fewer services such as pet hotels. The client record software is the least used 66.60% along with the least number of pet hotels with the ratio of 44.40%, showing the selective nature of the services offered. Pet product sales are still high with the ratio of 88.90%. Only 22.20% of clinics track financial status, and 55.60% manage their stock control. For profit margins 33.30% of clinics place in lowest tertile, while 33.30% clinics place in highest tertile in terms of cost to income ratios.

## 4. Discussion

### 4.1. General veterinary clinic characteristics and current problems

The veterinary clinics reflect a variety of socio-economic and operational features that could capture dynamics in urban veterinary practices. In our study, average duration of experience for clinics is about 10.43 years, supported by a mean staff size of 3.37 employees indicating that most clinics are well established in the market by durable provision of services for longer periods. In one study the authors found that young vets do not see the necessity to balance treatment with client’s requests which refers to spending time and gaining experience before actually handling clients [28]. Similarly, it was stated by [29] that outside skills and capabilities, clients demanded professionals when it came to the solving and decision making aspects. This would mean that this experience in the profession could suggest a sort of trust and loyalty from the clients, which is vital for keeping business alive in the competitive environment.

Further, the educations pursued by veterinarians include mostly Master’s degree with the ratio of 48.57% and a few Ph.D.s with the ratio of 17.14% in our study. While [1] reported the rate of veterinarians with a doctorate level of education as 14.2% in their study, the rate of veterinarians with postgraduate education was determined as 25% in the study conducted by [6]. This may mean highly competent work force and perhaps an indication that the clinics stress continuing education as a means of achieving high standards of services. For example, [30] further stress that education of veterinary staff on quality assurance and standards of laboratories is very necessary. Among the important features that ensure the diagnostic services offered by veterinary clinics are both accurate and reliable. This attention to quality directly contributes to the standard of care provided to clients and their pets.

The income analysis indicates that the source of major incomes is general examination followed by vaccination-parasite treatments, operations, and pet shop sales. In one study, [31] mentioned that preventative medicines are the main source of income in UK small animal practice. While vaccination and medicine costs are the greatest among all mentioned (30.28%), even labor and equipment costs (23.88% and 11.09%, respectively) account for a big portion of overall expenses. In their study, [6] observed that labor costs were in the first place among the cost items with 24.77%.

The client profile is dominated by cat patients, considering that 61.21% of the patients were cats, in comparison with 31.90% being dogs, which meets the urban pet-keeping trend to have smaller and more independent animals such as cats due to constraints in space and lifestyle. This breakdown affects service offerings and sources of income, since the income comes from general examinations and vaccination-parasite treatments currently represents the highest contribution.

The high reliance on general examination and vaccination-parasite treatments income points to a stable but probably limited revenue stream, as such services are more routine and hence predictable, as compared to specialized operations. This stable income in clinics is generated by the pet owners who are generally willing to invest in preventive care, since they are aware of long-term benefits of keeping their pets healthy [32]. On the other hand, the increasing costs of medicines and supplies make this model not very sustainable, and thus press clinics to find additional sources of incomes. The fact that 60% of the clinics within the scope of our study have pet hotel services and 88.6% of them sell pet products in their clinics proves that they need additional income sources.

However, such clinics that can strike a balance between routine and specialist services provided are more likely to be financially healthy in the long run. According to [33], the nature of the services imparted is a major determinant of the economic performance of the veterinary practices. Those practices that were able to provide a mix of routine and specialized care managed their resources optimally, and hence better economic performance was noted.

The operational challenges included unfair competition among clinics (31.40%), an increase in prices of supplies (11.40%), and problems with clients (14.30%). Professional undervaluation and staff shortage add to poor performance of the clinics. For this reason, to overcome these professional undervaluation and personnel shortage problems, veterinary clinics must be willing to adopt modern differentiating and cost management policies. For example, leveraging technologies in the provision of telehealth services can provide ease of access while optimizing available personnel [34]. As [35] express, the curriculum should prepare students to practice efficient telehealth given the current shortage of human resources. This way, on-site pressures may be reduced with simultaneous expansion of service provisions.

### 4.2. K-means cluster characteristics

A total of 43 omnibus tests were conducted, and no multiplicity adjustment was applied across the complete set of omnibus P values, increasing the risk of Type I error. Accordingly, the cluster comparisons were interpreted as exploratory and hypothesis-generating, with greater emphasis on coherent patterns across related operational, managerial, and financial variables than on isolated P-values. For descriptive purposes, post hoc power estimates were ≥ 0.80 for most statistically significant tests, with a minimum value of 0.753. However, these estimates were interpreted with caution and not used as evidence of the robustness or replicability of the findings, particularly given the small overall sample size and the limited number of clinics within each cluster. Moreover, given the relatively small overall sample size and the limited number of clinics within each cluster (ranging from 8 to 10), the resulting cluster profiles should therefore be regarded as indicative, data-driven groupings that require validation in larger, independent samples.

Cluster 1 has multiple services with average operational costs of 8616.77 $/month, which is significantly higher than the average operational costs of 2995.97 $/month in Cluster 4 (p < 0.005). This difference of course, may be explained by the wider service offering out of the clinics in Cluster 1, which included 90% of clinics offering pet hotels-, but also laboratory diagnostics that demand high investments in specialized equipment and staffing. The high laboratory income of $1029.18 in Cluster 1 versus $253.24 in Cluster 4 (p < 0.005) underlines the profitability of these additional services and indicates that resource use is strategically done in view of higher income opportunities. The potential of increasing incomes via new streams would apply to the clinics that extend their services, especially in a highly competitive market where price sensitivity is high [36].

Apart from these, staffing level also significantly influences the higher costs for the operation of Cluster 1 with 5.30 compared to only 2.33 staff in Cluster 4 (p < 0.001). Since they offer multiple facilities within their clinics and most of their services are diagnostic and surgical-related procedures, it is obvious that they require more staff. While these costs are high, they are counterbalanced by the highest monthly income ($10671.77; p < 0.005).

In contrast, Cluster 4 shows the low-cost operation model. This k-means cluster was characterized by the lowest monthly income with $5023.83 and low profitability at $2027.87. These are offset by the cost-effective principles, as testified by their average staff size of 2.33 and fewer high-income but high-cost services like diagnostic imaging and laboratory tests. Their focus on exotic animals may reflect a niche market strategy in an attempt to attain differentiation in a competitive environment, represented by 8.89% of the patients, which is the highest among all k-means clusters. They are, however, hindered from realizing higher profitability by a lack of diversified streams of incomes like pet hotels or grooming services. According to [7], the key opportunity for owners of companion animal clinic practices to take existing products and services to new markets is through the establishment or acquisition of additional practices. Theoretically, diversification can range from establishing boarding and grooming services to offering unrelated products and services to non-pet-owning clients.

Cluster 2, with moderately high operational costs amounting to $6900.04, stands between the k-means clusters of high-input and low-input models. The clinic in this k-means cluster prioritizes certain well-targeted high-demand services such as vaccinations that make up about 24.60% of their total cost. This concentration on vaccination perhaps could be due to the fact that prevention always needs a constant demand that produces regular income [32], which does not require large investments either in equipment or specialized personnel. The relatively high equipment-material costs (21.60%; p < 0.005) compared to Cluster 3 (4.44%) and Cluster 4 (7.81%) suggest a strategic choice to maintain modern equipment, perhaps to attract clients seeking reliable, up-to-date services.

Cluster 3 presents a strong strategic cost management with relatively low operational costs of $5178.43 and high profitability of $5093.65 (p < 0.001). It seems that these clinics focus on high-margin services such as vaccinations, at 42.78% of the costs (the highest among k-means clusters, p < 0.005), which do not need much investment while generating steady income. However, their rent costs are the highest among all k-means clusters (19.61%; p < 0.005). In a study, [37] emphasized that the share of rent cost in veterinary clinics varies according to criteria such as the region where the clinic is located, city or rural, the number of veterinarians and total income. Our findings suggest that these clinics are mostly located in districts with higher demand and higher income population (50% in Çankaya and 25% in Yenimahalle) where they take the advantage of location and turns into profit. Because 87.50% of the clinics in Cluster 3 are also in top tertile in terms of profit margin.

By contrast, the clinics in Cluster 3 have extended weekly working hours. With an average estimation of 81.75 working hours per week, clinics were able to rake in an average revenue of $5093.65. However, this extra workload can increase the risk of vet burnout [38], which severely affects emotional stability, professional job satisfaction, and high job turnover [39]. Such issues may have long-lasting effects, resulting in the workplace not being as productive in the long run. Instead of excessive working hours, rotational employee schedules and hiring additional workers may be preferred. This would not only help clinics make more revenue but also help maintain a satisfactory work-life balance.

In terms of financial indicators, Clusters 1 and 2 showed balanced cost management, with the majority falling in the second tertile, indicating controlled expenses compared to income. In contrast, Cluster 3 was very efficient, having a large share in the first tertile of the cost-to-income ratio, combined with higher labor productivity. This may have been due to better utilization of staff and an appropriate patient turnover ratio. At the same time, Cluster 4 shows higher costs and lower productivity, likely due to fewer services offered and underdeveloped financial-tracking mechanisms, since it had fewer clinics monitoring financial status (22.20%). Financial behaviors and awareness are important for understanding how to manage finances effectively among veterinary professionals. Those veterinary clinics that can clearly identify their financial goals and maintain a budget will be better positioned to navigate through the complexities of their operation and ensure their financial viability [33,40].

Further differences in financial efficiency between k-means clusters are underlined by the analysis of profit per patient and ATR. Indeed, Cluster 3 had a 75% proportion of clinics in the third tertile with regard to profit per patient, which reflects that this k-means cluster had the greatest ability to gain substantial profit relative to the patients served. This may relate to an increased emphasis on value-added services such as vaccinations and surgery, which account for a very large proportion of income [31]. Regarding ATR; Cluster 3 delivered the best, with 50% of its clinics in the third tertile, indicating successful asset utilization in income realization. Cluster 4 is behind, with most of the clinics in the two lower tertiles; this perhaps is because of underutilized investments and narrow diversification into streams of income, thus recommending the use of better asset management strategies for better financial outcomes and being competitive in the market [17].

However, in addition to revealing financial performance, it is also necessary to discuss why k-means clustering was used in this study. This analysis allowed us for a deeper understanding of business dynamics and intervention approaches by clarifying the various paths to financial success. Clinics classified under Cluster 3 maximize financial performance through effective operational processes and high-margin services, including vaccinations. On the other hand, clinics located in Cluster 1 counteract high operational expenses through increased volumes of laboratory tests and pet hotel services. Consistent with prior studies, these observations confirm that an integration mechanism combining specialization with diversity in incomes is important for veterinary clinic financial viability [36].

Similar to our findings, previous studies have also emphasized that operational efficiency, service diversification, and cost management are among the major determinants of veterinary clinic profitability [11,33]. Baguley [7] reported that diversified service models contribute positively to the long-term financial sustainability of companion animal clinics. In our study, particularly Cluster 1 clinics with diversified services and Cluster 3 clinics with stronger cost-efficiency structures demonstrated comparatively better financial performance, which is consistent with these previous findings.

On the other hand, for those caring for exotic animals, Cluster 4 shows low service diversification and low financial stability. The adaptive strategy required might be service expansion or identification of strategic partners. Cluster analysis thus provides a structured framework in strategic planning that would assist such clinics toward identifying strengths, mitigating risks, and refining business models toward better financial resilience.

## 5. Conclusion

This study provides an important framework for operational strategies and economic performance by deeply analyzing the socio-economic and financial characteristics of companion animal clinics. The research proceeds through four k-means clusters. Cluster 1 has the advantages of a multi-service model with high incomes, but faces high operational costs. Cluster 3 reveals how cost-effective strategies can result in high profitability. K-means cluster analysis revealed a balanced model in Cluster 2, with moderate costs and stable incomes, while Cluster 4 was focused on niche services with lean operations but had some problems regarding sustainable profit attainment. These highlight several ways in which companion animal clinics manage to adapt according to clients’ needs and market demands, and the framework will assist in assessing and improving clinic management.

This research underlines the importance of the alignment of service offerings with financial goals, therefore emphasizing the trade-offs between diversification, specialization, and efficiency. These findings would therefore certainly permit policymakers, along with veterinarians, to develop effective strategies which aimed at boosting the financial outcome as well as problems linked to cost rise along with growing competition. Other regions and additional variables may be included in this framework in future studies, such as those on client satisfaction, in order to better understand the performance of the clinics.

### 5.1. Limitations

Among the limitations of this study is its restricted geographical scope, which might limit generalizability in other regions with different economic and veterinary landscapes. This study also relies on available financial and socio-economic data, which might not represent all the drivers of success in the clinic, like client satisfaction and quality of service. In addition, since the data were collected largely before the COVID-19 pandemic, the findings primarily reflect the pre-pandemic operational and financial landscape of companion animal clinics. Future studies should use broader data sets and expand the scope of the study.

Another limitation is that revenues from vaccination and antiparasitic services were collected as a combined category rather than separately. Consequently, the individual contribution of these two important revenue streams to clinic profitability could not be evaluated independently.

The statistical findings should also be interpreted in light of the small sample size, the data-driven construction of the clusters, and the number of omnibus comparisons performed. Conducting 43 omnibus tests without adjustment across the complete family increased the possibility of Type I error, whereas the limited number of clinics may also have reduced the ability to detect some genuine differences.

The post hoc power estimates were considered descriptive only and do not establish the robustness or replicability of significant findings.

These results should therefore be interpreted as exploratory evidence, and future studies with larger samples are encouraged to confirm the cluster profiles identified here.

## Supporting information

S1 Fig Flowchart of the statistical framework. (TIFF)
